# The patient-safety implications of AI-based communication with migrants in general practice: a scoping review

**DOI:** 10.3399/BJGPO.2025.0107

**Published:** 2025-12-19

**Authors:** Anne Cronin, Anthony Kelly, Michelle Wrona, Patrick O'Donnell, Ahmed Hassan, Tonya Myles, Tadhg Fallon, Anne MacFarlane

**Affiliations:** 1 Participatory Health Research Unit, School of Medicine, University of Limerick, Limerick, Republic of Ireland; 2 Department of Electronic and Computer Engineering,, University of Limerick, Limerick, Republic of Ireland; 3 School of Medicine, University of Limerick, Limerick, Republic of Ireland; 4 Doras, Limerick, Republic of Ireland; 5 Cairde, Dublin, Ireland; 6 Department of Health, Government of Ireland, Dublin, Republic of Ireland

**Keywords:** primary health care, general practice, migrant health, interpretation, translation, artificial intelligence

## Abstract

**Background:**

Access to interpreters for refugee and migrant patients that do not share the same language and culture as their GPs is considered a critical healthcare adaptation. However, interpreters are not routinely available in many healthcare settings and artificial intelligence (AI) is increasingly used as a pragmatic alternative. The patient-safety implications of relying on AI for this purpose are under-researched.

**Aim:**

To identify and map available evidence on AI-facilitated synchronous communication between refugee or migrant patients and their healthcare provider, focusing on the patient-safety implications.

**Design & setting:**

A six-stage scoping review was undertaken, examining the international literature.

**Method:**

A literature search of five relevant electronic databases and grey literature from July 2017 to October 2024 was conducted. Data were extracted and synthesised accordingly.

**Results:**

A total of 220 articles spanning various healthcare contexts were screened, with five articles meeting inclusion criteria. These studies report use of the AI-tool Google Translate to address language barriers across diverse clinical settings, despite Google Translate not being designed to support synchronous communication or communication in medical contexts. Negative experiences of using these tools were reported more than positive experiences. Clinicians discussed specific concerns about reliability of Google Translate for medical terms, patient consent, and complex consultations.

**Conclusion:**

There is no evidence that using Google Translate to synchronously communicate medical information to refugees and migrants has been tested for patient safety, highlighting potential for translation inaccuracies impacting patient safety. In clinical settings, where the high stakes of failure are ever-present, such inaccuracies can result in misdiagnosis, inappropriate treatment, and serious harm.

## How this fits in

Artificial intelligence (AI) translation tools are increasingly used by refugees, migrants, and health professionals to communicate in primary care settings, in some cases being used as an alternative to trained interpreters. Many of these tools, including Google Translate, are not specifically designed to facilitate synchronous communication in medical settings. Clinicians in this study report scenarios where they use Google Translate reluctantly, owing to concerns about its accuracy. Some clinicians continue to rely on untested and unvalidated AI-powered tools.

## Introduction

Approximately 1 billion people worldwide are migrants, having journeyed within their own state or across a border from their usual place of residence, accounting for roughly one-eighth of the world’s population.^1^ It is currently estimated that approximately 43.7 million of these migrants are refugees.^2^

Migration has extensive public health implications, influencing healthcare accessibility, availability, acceptability, and service delivery.^3^ General practice has been pivotal in addressing health inequities within migrant populations, serving as a primary point of care and intervention.^4^ However, engaging with patients with limited language proficiency in the language spoken in the resettlement country, is an increasing issue for GPs working in these settings.^5,6^ Healthcare delivery can be substantially compromised if the ‘gold standard’, that is, a trained interpreter, is not provided in a cross-cultural consultation where the GP and patient do not share the same language or cultural background.^7–9^ These compromises include incomplete information exchange, inappropriate diagnosis and treatment, medication error, missed opportunities, and lower levels of patient trust.^10^ The evidence also tells us that healthcare costs are higher owing to inefficient use of resources, that is, repeat appointments and unnecessary tests for refugees and migrants.^11–13^

In the context of uneven implementation of trained interpreters in general practice, informal communication supports, such as family members, friends, and digital translation tools, are relied on.^12,14^ This practice reinforces existing concerns about patient safety and the discriminatory impact of substandard language interpretation.^15^ The use of AI-powered tools, including large language models, such as Google Translate and ChatGPT, has received enormous attention^16^ and their integration in health care in general has been met with a mixture of excitement over the potential to revolutionise patient care and trepidation about ethical, safety, and equity challenges.^17^

AI is commonly used as an umbrella term for various technologies, with their definitions and healthcare applications outlined in Table 1.

**Table 1.** Definitions of key AI technologies and their application in health care

Term	Definition	Key points	Use in healthcare settings
**Artificial intelligence (AI**)	The field of study focused on developing systems that can reason, learn, perceive, and act rationally in response to their environment^44^	Generates rational inferences, optimises outcomes, and adapts to circumstances^44^	Increasing use across healthcare settings, particularly medical imaging^45^
**Machine learning (ML**)	Subset of AI, using algorithms and statistical models to improve performance on tasks through experience, without explicit programming for each task^46^	Enables AI adaptability by learning from data, identifying patterns, and making predictions without explicit programming	Research, disease diagnosis, clinical decision support^47^
**Deep learning (DL**)	Subset of ML using artificial neural networks with multiple layers to analyse large datasets and make predictions or decisions^48^	The multiple layers represent a hierarchy of concepts, where higher layers represent more abstract features built on simpler ones	Deep learning has demonstrated considerable potential in areas such as clinical imaging, mining electronic health records, genomics, and mobile health^49^
**Machine translation (MT**)	Uses software, often based on statistical, rule-based, or neural network models, to automatically translate text or speech between languages without human intervention^50^	Rule-based machine translation (RBMT; 1950s –1990s): symbolic AI using predefined rules Statistical machine translation (SMT; 1990s–2010s) to provide more flexibility in translation based on data instead of pre-defined rules Modern MT (NMT): deep learning transformer models that capture long-range dependencies and context across entire sentences more effectively,^51^ trained on vast multilingual corpora, they have considerably improved translation accuracy and fluency across major languages, although performance still lags for minority and low-resource languages^52^ Google Translate: one of the most widely used NMT applications, leveraging transformers and vast multilingual datasets to provide real-time translation	Patient communication for clinical and health literacy, for example, discharge instructions
**Large language models (LLM**)	Type of DL trained on vast text datasets and using transformer models, designed to understand and generate natural language^53^	Revolutionised NLP and enabled a wide range of language-related tasks, including language translation	Clinical text analysis and decision support^54^
**Natural language processing (NLP**)	Branch of AI focused on enabling machines to understand, interpret, and generate human language using ML, DL, and linguistic analysis^55^	Processes and analyses natural language data, a core component of translation tasks	Electronic health record data extraction, patient communication

While in-person and remote interpreter implementation challenges persist, GPs are endeavouring to respond to the problem by integrating AI-powered technology into their consultations with patients who do not share their language.^6,18^ Evidence about the patient safety implications of AI use in general practice settings is lacking.^16^ Consequently, this scoping review sought to examine patient safety implications of using AI-powered tools to support synchronous communication across diverse clinical settings.

## Method

Scoping reviews play an important role in knowledge synthesis, mapping the breath and scope of emerging areas of research interest.^19^ This scoping review systematically maps existing research, including both qualitative and quantitative studies and grey literature. The methodological approach was guided by Peters *et al* ^20^ and Colquhoun *et al,* ^21^ building on Arksey and O’Malley’s six-stage framework.^22^

### Identifying the research question

This inquiry emerged from discussions with health policymakers, healthcare providers, and non-governmental organisation (NGO) colleagues regarding the role of AI as a potential solution in scenarios where interpreters are unavailable or challenging to implement. While the primary interest was AI use as a communication support in GP consultations in primary care settings, early piloting indicated a small literature, therefore the research question was broadened to search all healthcare settings. Table 2 is a breakdown of the population, intervention, comparison, outcomes, and context (PICOC).

**Table 2.** Research questions derived or framed according to the PICOC^56^

Population	Patients and healthcare providers who meet and do not have shared language and cultural background, that is, who meet in a cross-cultural consultation
Intervention and issue	Use of AI tools to support communication in cross-cultural consultations
Comparative intervention	On-site, video, or phone medical interpreters supporting communication in cross-cultural consultations
Outcomes	Patient safety; accuracy and associated risks of using AI-powered tools to communicate
Context	Studies investigating use of AI in cross-cultural consultations; July 2017– August 2024

### Identifying relevant studies

Studies that reported empirical data on the use of AI for medical interpreting in synchronous consultations either in-person or remotely with refugees and migrants and published between July 2017 to August 2024 were included. Articles were excluded if they reported the use of AI for health literacy, health monitoring, processing tests, or the use of AI in asynchronous consultations with refugees and migrants.

The interdisciplinary team, assisted by the University of Limerick (UL) librarian (PP), conducted an electronic database search of PubMed, Scopus (includes MEDLINE, Embase), CINAHL,IEEE Xplore and ACM (Association for Computing Machinery) (see Table 3 for search terms and syntax). Grey literature searches were conducted using Google and further reference chaining using the AI-powered citation-based literature mapping tool ResearchRabbit and Undermind, an AI-powered research assistant. Searches were also conducted on the social media platform X (formerly Twitter), using the search term #medicalAI. Grey literature searches continued to October 2024.

**Table 3.** Search terms and syntax

AI	Communication	Interpreter, mediator, translator	Health	Refugee, migrant
Artificial intelligence OR app OR machine learning OR Chatbot OR large language model OR natural language processing OR conversational agent OR ChatGPT	communicat* OR interpret* OR translat* OR mediat* OR intermediary	language OR cultur* OR cross-cultural OR multicultural OR transcultural	Health	asylum* OR refugee* OR migrant* OR migrat* OR emigrant* OR emigrat* OR immigrant* OR nomad* OR foreigner* OR displaced OR stateless OR state-less OR noncitizen* OR non-citizen* OR outsider* OR newcomer* OR "newly arrived" OR "new arrival*" OR "recent entrant*" OR "non national" OR non-national OR ethnic

### Study selection

The Covidence screening tool was used for title and abstract screening by the following three researchers: AC, AK, MW. Full-text screening was conducted by AC, AK, MW, AMF, and POD and conflicts were resolved by AC and AMF.

### Data charting

A summary of information including description of AI used to communicate with refugees and migrants, use of AI in synchronous consultations, reports of patient safety or clinical risk were recorded. Critical appraisal of the selected studies was conducted using the Critical Appraisal Skills Programme (CASP) checklist criteria for qualitative studies,^23^ see Supplementary File 1.

### Collating, summarising, and reporting results

Aligned with the guidelines in the *JBI Manual for Evidence Synthesis,* ^20^ we conducted a narrative synthesis that maps our findings to the central research question. Each included study is classified and examined based on its reporting of AI-powered tools facilitating synchronous communication in healthcare settings and reporting of patient-safety implications.

### Consultation with expert stakeholders

Data analysis and interpretation of results was enhanced by the involvement of colleagues from general practice (POD), the Department of Health, the Republic of Ireland (TF), and refugee and migrant NGOs operating in the Republic of Ireland: Doras and Cairde (AH, TM). This further validated our findings. It provided important perspectives and insider knowledge^22^ from the field of refugee and migrant health policy and advocacy about AI use, in response to ongoing communication challenges experienced by refugees, migrants, and health professionals.

## Results

### Search results

**Figure 1.**
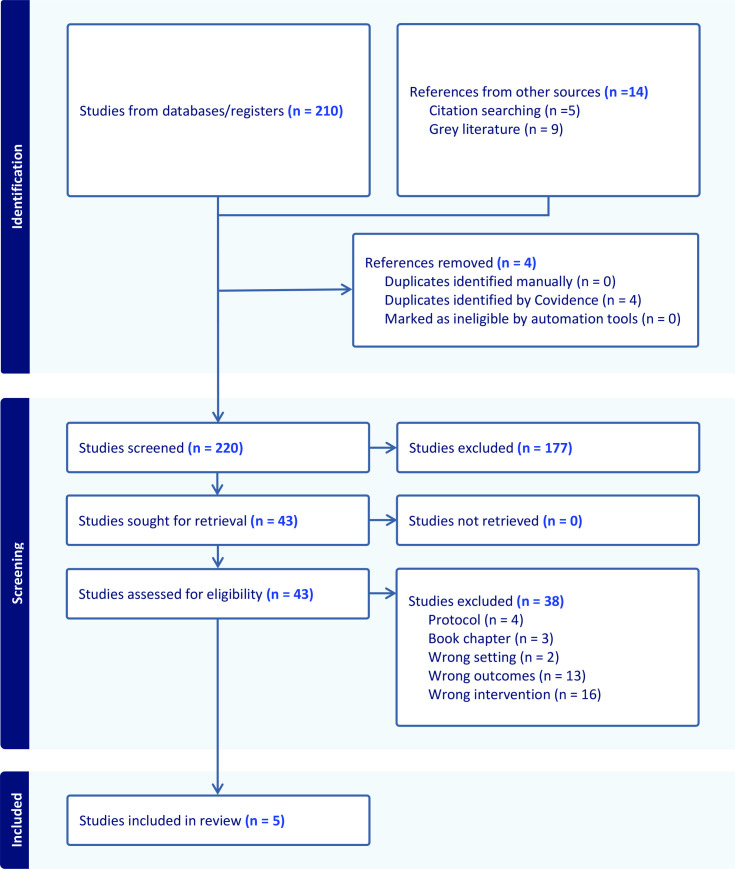
Preferred Reporting Items for Systematic reviews and Meta-Analyses (PRISMA) flow diagram

Our search yielded 220 studies. After title and abstract screening, 43 articles were fully screened, with five included for final review. We report our results according to the Preferred Reporting Items for Systematic review and Meta-Analyses extension for Scoping Reviews (PRISMA-ScR), shown in Figure 1.^24^

### Study characteristics and settings

All five studies were peer-reviewed and published. Each referenced AI, either as a future conceptual tool or one currently supporting communication with refugees and migrants. Table 4 outlines the key characteristics of the five qualitative studies, conducted in North America, Europe, and Asia. Regarding data representation: 59% of the data are from clinical perspectives (*n* = 51), and 41% from migrants’ perspectives (*n* = 35).

**Table 4.** Study characteristics

Reference and author	Location	Participant group	Data collection methods	Topic
**1. Brown *et al* 2023** ^25^	France: immigration centres	Clinicians (11 doctors and nine nurses)	Qualitative interviews	Exploration of conceptual communication tool in HIV, hepatitis B, and hepatitis C screening
**2. Lindström and Pozo 2020** ^27^	Sweden: child healthcare centres and cultural interpreter centres	Clinicians (five nurses and four doulas)	Qualitative interviews	Digital solutions that assist maternal care for migrant mothers
**3. Tseng *et al* 2023** ^26^	Thailand: unspecified primary and secondary care	Migrants (10 Thai workers) and stakeholders (two clinicians and seven non-clinicians)	Qualitative interviews	How migrant workers seek health care and communicate with health professionals
**4. Mehandru *et al* 2022** ^28^	US: private practice, county hospitals, community hospitals, and academic institutions	Twenty clinicians (physicians, surgeons, nurses, and midwives working in cardiology, orthopaedic surgery, nephrology, family medicine, obstetrics and gynaecology, trauma surgery, and emergency medicine)	Qualitative interviews	Language barriers experienced by migrant patients and the use of machine translation to assist
**5. Liebling *et al* 2020** ^29^	US and India: unspecified primary and secondary care	Migrants (nine in the US and 16 in India)	Qualitative diary study and interviews	The usage, practices, and limitations of mobile translation tools for migrants with low language proficiency

### Evidence of AI use in synchronous conversation

Chatbots simulating human-like conversations through automated text or voice are mentioned in Brown *et al* ^25^ and Tseng *et al* ^26^ as conceptual solutions to engage hard-to-reach patients and support migrant communication. However, neither reported actual chatbot use supporting multi-turn synchronous conversations. While there is growing interest in AI tools for ‘*simultaneous translation*’ as imagined in Brown *et al* ^25^ ‘*You just put your language in, and then when you are speaking, it translates immediately*’, our review found no evidence of existing AI applications replicating in-person interpreters. Generic AI-powered tools are not included in any evidence-based clinical interpretation framework identified in this review. However, we noted growing literature on AI tools supporting asynchronous communication, built on pre-set phrase banks.

### The role of Google Translate

The most frequently reported AI tool for communicating with migrant patients across healthcare settings is Google Translate. Studies did not specify whether the free or paid version was used. Clinicians reported using Google Translate in ‘*lower-stake interactions*’ such as follow-up home visits (Lindström and Pozo),^27^ conversations about screening (Brown *et al*),^25^ and history-taking (Mehandru *et al*).^28^ Migrants used it for language learning (Liebling *et al*)^29^ and finding images to explain symptoms (Tseng *et al*).^26^ Most clinicians in Mehandru *et al* ^28^ used Google Translate when they had partial knowledge of the patient’s language but needed support with specific words or phrases.

GPs and patients said their use of Google Translate is influenced by previous experiences of working with interpreters, leading to concerns around confidentiality (Brown *et al),* ^25^ difficulty navigating the dual role of cultural mediator and interpreter, unplanned interpreter requests, problematic phone interpretation, patient preference for informal supports (Lindström and Pozo),^27^ concerns about quality of available interpretation services (Tseng *et al*),^26^ time constraints, mistranslations, resource limitations, cultural taboos (Mehandru *et al),* ^28^ and lack of access to in-person interpreters (Liebling *et al*).^29^

Positive implications were strongest in Lindström and Pozo:^27^ midwives and doulas felt it aided communication with migrant mothers via shared images and videos and supported integration into Swedish society. Other studies briefly noted benefits of using Google Translate such as ease of use (Brown *et al*)*,* ^25^ usefulness when clinicians were partially familiar with a language (Mehandru *et al*)*,* ^28^ and language learning support (Liebling *et al*).^29^

However, most studies noted challenges with Google Translate, including inaccuracies, failure to capture dialectal or contextual nuance, and lack of support for low-literacy users (Mehandru *et al*)*.* ^28^ Limitations arise primarily because it is not designed for medical use and may not recognise medical terminology, increasing the risk of errors (Tseng *et al*).^26^ Although Google Translate has a ‘conversation function’ (Liebling *et al*),^29^ it lacks capability for complex, multi-turn conversational translation. Liebling *et al* ^29^ reported further complications, as the responsibility for the use of Google Translate often falls on the patient.

### Patient safety

Three of the five studies discussed patient safety. Tseng *et al* ^26^ reported migrant concerns about potential harm if chatbots are not designed and deployed with the relevant and appropriate guardrails, emphasising the need for patient-focused, transparent, secure designs. Migrant responders in Liebling *et al* ^29^ discussed risks associated with inaccurate medical terminology and stressful experiences navigating device-mediated multi-turn conversations.

Mehandru *et al* ^28^ examined safety from clinicians’ perspectives, focusing on the lack of governance and validation of AI translation tools such as Google Translate. It noted the ‘*high stakes of failure*’ at the intersection of machine learning and health care and that in these instances, not providing a translation is often better than providing an incorrect one. The researchers suggest that these risks could be reduced by institutional or medical board validation and call for more transparent safety standards built into machine translation.

However, Mehandru *et al* ^28^ also found that clinicians often prioritise effective communication (cultural appropriateness and trust-building) over perfect accuracy, as it improves patient outcomes. It also noted a pervasive ‘yes’ culture, more pronounced with language barriers; for example, a patient appeared to understand a GP but later expressed doubt to a nurse. Concerns about taking too much of the clinician’s time or lacking the words to express doubts may explain such behaviour. Some clinics sought to address these limitations by employing cultural navigators, distinct from medical interpreters.

## Discussion

### Summary

This scoping review identifies the following five key findings: an absence of evidence about AI-powered tools used to support synchronous communication in primary care; a lack of any evidence that an AI-powered tool can support synchronous interpretation safely; evidence that Google Translate is being used in healthcare settings; reliance on Google Translate amid concerns about real-life interpreter availability and use; and predominantly negative perceptions of its use.

### Strengths and limitations

This interdisciplinary scoping review of international literature facilitated a comprehensive synthesis of evidence from research at the intersection of AI and cross-cultural GP consultation, conducted since 2017. The team combined database searching, reference chaining, and a comprehensive search of grey literature to maximise the sample of included articles.

A notable limitation of this review is the restriction to studies published in English and the risk of missing data in a rapidly evolving field.

### Comparison with existing literature

In line with previous reviews,^30^ there is a dearth of evidence about AI-powered tools for communication in health care. Of the studies included in this review, none report specifically from general practice, which is a significant evidence gap as GPs are a pivotal healthcare provider in resettlement countries and are using Google Translate. A recent study examining the use of interpreters in Irish general practice found that 61% of GP participants use Google Translate to support interpretation with patients with limited English proficiency.^6^ A 2024 Dutch study found that GPs (and paediatricians and infectious disease specialists) continue to rely on Google Translate to communicate with migrants who visit a consultation alone.^31^ These findings are substantiated by community partners in this scoping review who report frequent use of Google Translate in GP consultations with refugees and migrants, notwithstanding a strong patient preference for in-person interpreter supports. Persistent use of Google Translate risks replacing scheduled interpreter bookings. This can lead to the diminished use of human-trained interpreters and a potential over-dependence on AI solutions, which are not designed for medical interpretation. It also potentially presents a concerning instance where a general-purpose AI technology is being rapidly integrated into the healthcare sector to an unprecedented extent. This is deeply problematic given the lack of regulatory oversight, leaving questions about data security, patient consent, and clinical accountability,^32–35^ which are not explicitly addressed in the European Union AI Act’s risk-based taxonomy.^36^ The UK government plans to introduce legislation to address AI risks later in 2025.

The available evidence in this review reveals a reliance on Google Translate as a substitute for in-person interpretation, even as those using it, report ongoing concerns regarding the legal and clinical risks associated with relying on these technologies. Some acknowledge that such tools are preferable to having no communication support at all, while others take the view that no interpretation is sometimes preferable to inaccurate interpretation. These findings are in line with previous reports that machine translation tools are not capable of replacing interpreters in healthcare communication but are useful as a complementary support in synchronous communication with low-risk contents^37^ and that the translation quality of Google Translate required post-editing as a mandatory step before reaching the end user, that is, the patient.^38^ Another contemporary study that developed a framework for clinical use of large language models in patient interactions, found that AI continues to face challenges in facilitating natural conversations and advises that healthcare professionals continue to collect patient data in the first instance.^39^

Problems with interpreter availability, the time it takes to access an interpreter, or concerns about introducing a third party into the consultation are influencing the use of Google Translate in health care. This underscores the importance of intensifying efforts to investigate ways to optimise implementation of trained interpreters in general practice and other healthcare settings.

### Implications for research and practice

In light of the gaps presented, further research focused on communication scenarios in general practice, in respect of both real-life interpreters and interpreter applications is recommended. We suggest that potential to cause patient harm is given careful consideration. To mitigate potential for harm, Omiye *et al* argue that at a minimum, larger-scale quantitative studies are necessary to examine patient-safety implications, before the widespread implementation of AI technologies.^40^ This would generate essential, measurable data on the performance of machine translation in clinical settings, support the generalisability of findings across diverse patient populations and healthcare contexts, and enable the identification and statistical analysis of risks from AI-generated errors compared with those made by human interpreters. Further evaluation using patient-reported outcome measures could offer key insights into the impact of language barriers in cross-cultural consultations with refugees and migrants.^41^

In terms of what clinicians and healthcare organisations can do now to mitigate the risks posed by the use of clinically untested AI-powered tools, such as Google Translate, we suggest that providers strengthen both the availability and utilisation of trained interpreters through capacity building, workforce planning, and targeted staff training, underpinned by a commitment to consistent application of best practice. Simultaneously, we advise that clinicians and practice managers carry out detailed risk assessments for any AI tool used to communicate with patients, identifying possible failures, biases, and misuse, and put in place control measures to mitigate these risks while discontinuing the use of AI-powered tools not specifically designed, developed, or validated for medical interpretation. It is crucial that this process involves informing patients when AI is used in their care and always seeking consent.

AI tools (such as those reported in this review) demonstrate many limitations in performing or responding to nuanced and complex interactions between patients and GPs,^42^ which is a considerable concern in respect of unique and complex needs of refugees and migrants, particularly those who have experienced a traumatic migration experience. These circumstances could necessitate sensitive, context-aware communication strategies that extend beyond basic language translation to ensure compassionate and patient-centred care. Health policy and service planning must take these considerations into account to minimise health inequities.^43^

To support safe and effective patient care, the following principles are recommended for cross-cultural consultations with refugees and migrants:

- Clinicians have a responsibility to ensure best practice is followed, including providing access to trained interpreters across all modalities, such as in-person, video, and telephone.
- Clear, consistent protocols for accessing trained interpreters should be available and visible across all healthcare settings.
- In light of current evidence, the use of AI-powered tools not specifically designed, developed, or validated for medical interpretation should be discontinued.

## Data

The dataset relied on in this article is available from the corresponding author on reasonable request.
